# Treatment with αvβ3-integrin-specific 29P attenuates pressure-overload induced cardiac remodelling after transverse aortic constriction in mice

**DOI:** 10.1016/j.jmccpl.2024.100069

**Published:** 2024-03-12

**Authors:** Alexandra Njegić, Lina Laid, Min Zi, Eleni Maniati, Jun Wang, Alexandru Chelu, Laura Wisniewski, Jenna Hunter, Sukhpal Prehar, Nicholas Stafford, Chaim Gilon, Amnon Hoffman, Michael Weinmüller, Horst Kessler, Elizabeth J. Cartwright, Kairbaan Hodivala-Dilke

**Affiliations:** a Barts Cancer Institute, Queen Mary University of London, Charterhouse Square, London EC1M 6BQ, United Kingdom; b Division of Cardiovascular Sciences, School of Medical Sciences, University of Manchester, Manchester M13 9PT, United Kingdom; c Division of Diabetes, Endocrinology & Gastroenterology, School of Medical Sciences, University of Manchester, Manchester M13 9PT, United Kingdom; d Institute of Chemistry, The Hebrew University of Jerusalem, Jerusalem 91904, Israel; e Institute for Drug Research, School of Pharmacy, Faculty of Medicine, The Hebrew University of Jerusalem, P.O. Box 12065, Jerusalem 91120, Israel; f Institute for Advanced Study, TUM School of Natural Science, Technische Universität München, Lichtenbergstr. 4, 85747 Garching, Germany

**Keywords:** Heart failure, Integrin, Transverse aortic constriction, Cardiac hypertrophy, Cardiac fibrosis, RGD-mimetic

## Abstract

Heart failure remains one of the largest clinical burdens globally, with little to no improvement in the development of disease-eradicating therapeutics. Integrin targeting has been used in the treatment of ocular disease and cancer, but little is known about its utility in the treatment of heart failure. Here we sought to determine whether the second generation orally available, αvβ3-specific RGD-mimetic, ***29P***, was cardioprotective. Male mice were subjected to transverse aortic constriction (TAC) and treated with 50 μg/kg ***29P*** or volume-matched saline as Vehicle control. At 3 weeks post-TAC, echocardiography showed that ***29P*** treatment significantly restored cardiac function and structure indicating the protective effect of ***29P*** treatment in this model of heart failure. Importantly, ***29P*** treatment improved cardiac function giving improved fractional shortening, ejection fraction, heart weight and lung weight to tibia length fractions, together with partial restoration of Ace and Mme levels, as markers of the TAC insult. At a tissue level, ***29P*** reduced cardiomyocyte hypertrophy and interstitial fibrosis, both of which are major clinical features of heart failure. RNA sequencing identified that, mechanistically, this occurred with concomitant alterations to genes involved molecular pathways associated with these processes such as metabolism, hypertrophy and basement membrane formation. Overall, targeting αvβ3 with ***29P*** provides a novel strategy to attenuate pressure-overload induced cardiac hypertrophy and fibrosis, providing a possible new approach to heart failure treatment.

## Highlights

• ***29P*** improves cardiac function following transverse aortic constriction (TAC).
• Treatment with ***29P*** reduces cardiac hypertrophy and fibrosis post-TAC.
• RNAseq reveals ***29P***-mediated changes to metabolic, fibrotic and hypertrophic genes.

## Graphical abstract

**Figure:**
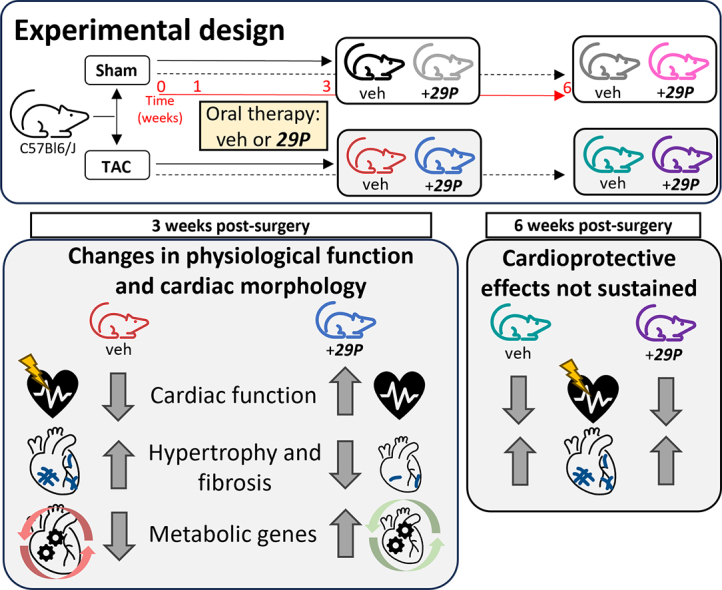

## 1 Introduction

Heart failure (HF), often the terminal aetiology of many cardiovascular diseases (CVD), is predicted to affect around 64 million people globally [1]. Targeting the progression of CVD is therapeutically challenging owing to the myriad of HF symptoms. In general, HF is often accompanied by poor cardiac function, deposition of non-contractile fibrotic tissue, an insufficient coronary microvascular and pathological cardiomyocyte hypertrophic remodelling [2]. Although advances in therapeutics have improved the diagnostic outcome of patients with HF, the 5-year mortality rate remains high. Therefore, there remains a need to develop novel therapeutics to improve outcome in patients with HF.

Integrins are heterodimeric, allosteric, transmembrane adhesion glycoproteins composed of a single α and a single β subunit [3]. Integrin αvβ3 is expressed on multiple cell types and is upregulated on the neovasculature; for example, within tumours and the myocardium post-myocardial infarction [[4], [5], [6], [7], [8], [9], [10]]. The upregulation of αvβ3/αvβ5 in proliferating vascular endothelial cells have led to the development of αvβ3/αvβ5 antagonists, such as the RGD-mimetic pentapeptide Cilengitide [[11], [12], [13]]. Cilengitide at its maximally tolerated doses (5 mg/kg) is anti-angiogenic, anti-adhesive and inhibits tumour cell proliferation in gliomas [[13], [14], [15]]. However, Cilengitide has hormetic effects and low-dose Cilengtide (ldCil) promotes angiogenesis *in vitro* and *in vivo*, a process termed vascular promotion [[16], [17], [18]]. Mechanistically, ldCil does not affect the adhesive function of endothelial cell αvβ3/αvβ5, but instead promotes the activation and recycling of the pro-angiogenic receptor VEGF-receptor 2 (VEGFR2) [17]. Exploiting this feature of ldCil, application in an abdominal aortic constriction (AAC) murine model of cardiac hypertrophy was shown to attenuate pressure overload remodelling and increase cardiac angiogenesis [16]. More recently, ldCil was also shown to improve re-perfusion following hindlimb ischaemia through enhanced neoangiogenesis, increased ischaemic muscle VEGF-A expression and promotion of macrophage infiltration and polarization [19].

Unfortunately, due to its failure to improve outcome in clinical trials for the treatment of glioblastoma the future of Cilengitide was hindered [20]. Cilengitide is administered intravenously and whilst its affinity for αvβ3 is subnanomolar (IC_50_ = 0.6 nM), it also has residual affinity for αvβ5 (IC_50_ = 8.4 nM) and α5β1 (IC_50_ = 15 nM) integrins [13,21]. To overcome these limitations, second generation RGD-mimetics were developed [21,22]. One such peptide, termed ***29*** [*c*(*vRGDA*A)] exhibits a favourable preference for αvβ3 (IC_50_ = 0.6 nM) over both αvβ5 (IC_50_ = 145 nM) and α5β1 (IC_50_ = 21 nM) integrins, docks to αvβ3 in a similar mechanism to Cilengitide and has been engineered to be orally available [21,23,24]. The orally available peptide prodrug ***29P*** [*c*(*vR(Hoc)_2_GD(OMe)A*A)] is cleaved into the active hexapeptide ***29*** in plasma [21]. *In vitro*, lower doses of ***29*** promote the upregulation of VEGFR2 protein expression in HUVECs and *in vivo* (50 μg/kg) it can increase angiogenesis of blood vessels within a subcutaneous Lewis Lung Cell tumour model similar to the effects of ldCil [21].

Here we show that ***29P*** can prevent adverse cardiac remodelling and restore cardiac function in a transverse aortic constriction (TAC) pressure-overload model of cardiac hypertrophy. RNA sequencing suggests that ***29P*** can modify key genes involved in cardiac hypertrophy, fibrosis and fatty acid metabolism, suggesting a new mode of action for ***29P***.

## 2 Materials and methods

### 2.1 Animals

Male C57Bl/6 J (Envigo) aged between 7 and 8 weeks and weighing between 21 g–28 g were subject to TAC using methods detailed previously [25,26]. Mice were housed in a pathogen-free, temperature- and humidity-controlled environment (21 ± 2 °C, light:dark cycle of 12 h) and were maintained in ventilated cages with access to standard chow diet and sterile water. The procedures described in this study were performed in accordance with the U.K. Animals (Scientific Procedures) Act (1986) and institutional guidelines for laboratory animal research at The University of Manchester, UK.

### 2.2 Transverse aortic constriction surgery

TAC was performed as detailed previously [25,26]. Mice were anaesthetised (3 % isoflurane) and given 0.1 mg/kg buprenorphine subcutaneously. Mice were intubated and ventilated (200 breaths per minutes, tidal volume of 0.1 ml [Minivent, Harvard Apparatus]) and the anaesthetic reduced (2 % isoflurane). A partial thoracotomy between the left second and third rib was performed and then a 27G needle was placed between the brachiocephalic artery and left common carotid artery which was ligated with a 7–0 prolene suture. The needle was removed to yield a constriction 0.41 mm in diameter. The chest and skin were then closed with a 6.0 prolene suture. Sham operated mice were subject to the same surgical procedure without ligation. All mice were given 0.1 ml/kg of saline intraperitoneally post-surgery. Upon the termination of experiment, mouse hearts were excised and drained of blood in sterile saline solution. Hearts and kidneys were dried, weighed and then roughly sectioned to provide tissue for RNA, protein analysis and histology. Lungs were excised and weighed and the tibia length measured to normalise the heart, kidney and lung weight.

### 2.3 Oral gavage treatment with *29P*

The oral prodrug ***29P*** was kindly provided to use by Chaim Gilon, Amnon Hoffman, Michael Weinmüller and Horst Kessler [21]. Mice were randomly assigned to receive either 6 doses of 50 μg/kg ***29P*** or volume-matched saline vehicle (Veh) as control, delivered through oral gavage. Treatment commenced 1-week following TAC surgery for 2 weeks with each gavage at least 48 h apart. Experiments were ended at two different time points following ***29P*** treatment: either at 3 weeks post-TAC or at 6-weeks post-TAC, *i.e.* 3 weeks after cessation of ***29P*** administration.

### 2.4 Transthoracic two-dimensional echocardiography

Mice were subject to echocardiography 1-, 3- and 6-week's post-TAC using the Vevo770 ultrasound machine and associated 14 MHz transducer (VisualSonics, FujiFilm). Mice were anaesthetised (1 % isoflurane) and heart rate maintained at 400–450 beats per minute. A parasternal short-axis M-mode image at the level of the papillary muscles was captured and used to estimate cardiac ejection fraction and fractional shortening (please see supplementary methods for calculations).

### 2.5 Histology

Full staining methodologies and analysis parameters are available in the supplementary methods. Briefly, heart mid-sections were fixed in 4 % paraformaldehyde, processed (Leica) and embedded in paraffin wax. 5 μm thick sections were obtained on poly-l-lysine coated slides. Both Haematoxylin and Eosin (H&E) staining and Masson's trichrome staining was performed using an automated stainer (Leica). Isolectin β4 (Vector) was used to visualise the microvasculature. Briefly, rehydrated slides were incubated with hydrogen peroxide block and then subject to sodium citrate (pH 6)-mediated antigen retrieval. Avidin and biotin blocking (Jackson) was performed and slides were incubated with biotinylated isolectin β4 (1:100; Vector). Slides were then incubated with streptavidin peroxidase (Abcam), developed using the DAB chromogen/DAB substrate method (Abcam) and counterstained with haematoxylin, dehydrated, cleared and mounted. All slide imaging was performed using the 3D-Histech Pannoramic-250 microscope slide-scanner (3D-Histech) and visualised using the Caseviewer software (3D-Histech).

### 2.6 Histological quantification

Cardiomyocyte cross-sectional area was obtained using Caseviewer (3D-Histech). A minimum of 100 transverse cardiomyocytes within the left ventricle (LV) were measured. The percentage of fibrosis within the LV was calculated using the semi-automated Caseviewer QuantCentre (3D-Histech) plugin. Fibre phenotypes were explored using The Workflow Of Matrix BioLogy Informatics [27] (TWOMBLI) plugin for ImageJ. Blood vessel images were cropped around longitudinal vessels and vessel length obtained using the FIJI (Image J) Skeletonize (2D/3D) [28] and Analyse skeleton plugin features [29]. Vessel length was then expressed as a percentage of each image area. An average of 3–4 images were used for statistical analysis.

### 2.7 RNA sequencing of left ventricle apex at the 3-week experimental endpoint

The full method for RNA extraction and sequencing is detailed in the supplementary methods.

#### 2.7.1 RNA extraction

RNA from snap frozen left ventricular apex tissue was extracted using the TRIzol (ThermoFisher)/chloroform method.

#### 2.7.2 RNA sequencing

RNA sequencing was performed by the genomics facility at Queen Mary University London.

RNA quantification was performed using the Nanodrop, followed by qualitative RNA analysis using the Agilent 2100 Bioanalyser according to manufacturer's guidance (Agilent). The mRNA Library was prepared using NEBNext Ultra II (New England Biolabs) and quantification of mRNA libraries was performed using Qubit 2.0 fluorometer. mRNA fragment size was checked using the Agilent 4200 Tapestation (Agilent). All samples were processed for individual Illumina next generation sequence library denaturation and loading. Following the sequencing run, 2 out of 20 samples had a read per million value of <10 million and were excluded. The data was aligned to the mouse genome (Mm10 genome assembly) using Partek Flow (Star 2.3.7), with an alignment rate of 98 %. Genes and transcripts were annotated with Genocode 25 and the number of reads within exons on average 84 %. Normalisation of data was performed using counts per million (CPM) + 0.0001.

#### 2.7.3 RNA-Seq data analysis

To identify differentially expressed genes (DEGs), normalised data was subject to DESeq2 differential gene expression analysis (*p* ≤ 0.05) and visualised using Venn diagrams. Enrichment of overlapping genes was performed with R package dnet using GOBP with significant terms identified (adjusted *p* ≤ 0.05). Annotated pathways were obtained from the Molecular Signature Data Base (MSigDB, v7.5.1). Furthermore, DEGs (*p* ≤ 0.05, ±0.3 Log_2_[CPM]) were inputted into STRING [30] for network analysis on Cytoscape [31], the largest interacting subcluster was subject to GOBP enrichment (adjusted p ≤ 0.05).

### 2.8 Single cell expression of Itgb3 in the heart following TAC

The publicly available Gene Expression Omnibus (GEO) database was searched for single cell RNA-Seq (https://www.ncbi.nlm.nih.gov/geo/). This identified the database GSE18072 (subseries of GSE180794 [32]) which contained data from endothelial cells, cardiomyocytes and fibroblasts at 1-week and 8-weeks post-TAC. The Log_2_[CPM] and the *P*-value were obtained from the spreadsheets available for download.

### 2.9 Statistics

All statistical analysis was performed using GraphPad Prism, each statistical test used is detailed within the figure legends (GraphPad, v9.2.0).

## 3 Results

### 3.1 *29P* treatment ameliorates pressure overload induced cardiac hypertrophic remodelling and impeded cardiac function at 3 weeks post-TAC but not post-*29P* treatment cessation

**Fig. 1:**
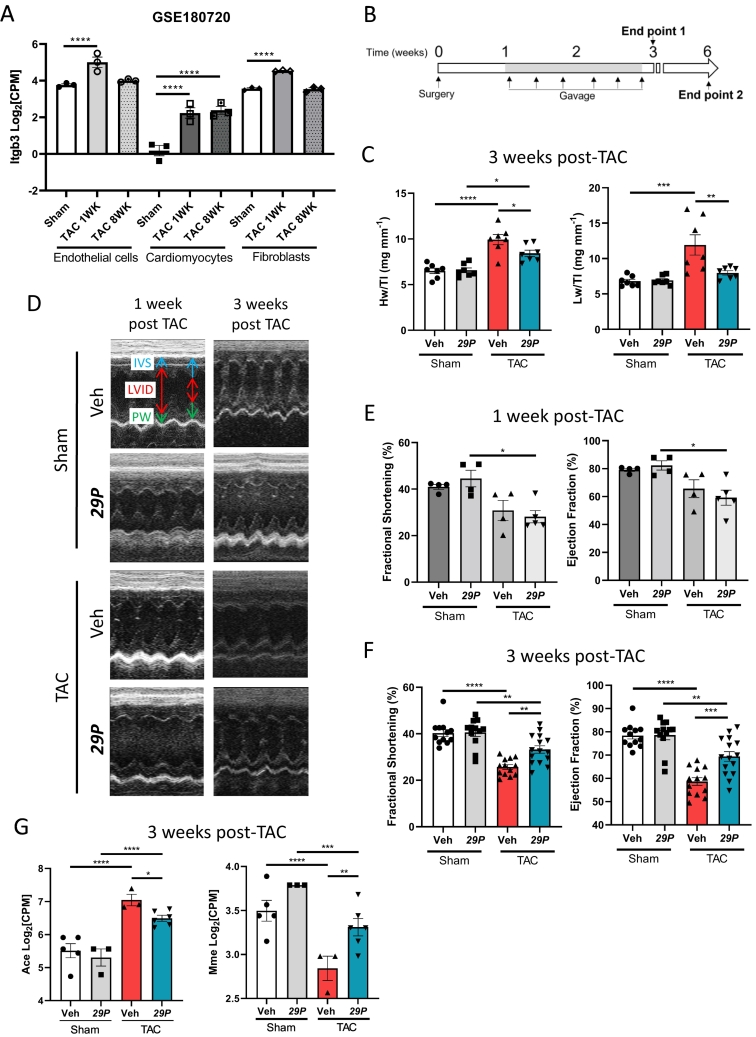
***29P*** confers protection against cardiac remodelling and improved cardiac function at 3 weeks post-TAC. A Expression of *Itgb3* in isolated cardiac endothelial cells, cardiomyocytes and fibroblasts, n = 3. B Timeline schematic detailing the onset of treatment and the two study endpoints utilised. C Morphometric parameters following 3 weeks TAC. ***29P*** treatment significantly reduces Hw:Tl and Lw:Tl ratios. Sham Veh n = 7, Sham ***29P*** n = 7, TAC Veh n = 7, TAC ***29P*** n = 7. Two-way ANOVA with Tukey's multiple comparison test (*p < 0.05, **p < 0.01, ***p < 0.001, ****p < 0.0001). D Representative short-axis M-mode traces at 1-, 3- and 6-weeks post-TAC (interventricular septum [IVS, blue], internal diameter [LVID, yellow] and posterior wall [PW, red]). E, F Quantification of fractional shortening (FS) and ejection fraction (EF) from echocardiography performed at (E) 1- and (F) 3-weeks post-TAC. E Retrospective analysis of echocardiography at 1-week post-TAC. Mice randomly assigned to receive ***29P*** had reduced cardiac function when compared to Sham controls. Sham Veh n = 4, Sham ***29P*** n = 4, TAC Veh n = 4, TAC ***29P*** n = 5. F EF and FS were reduced following TAC however ***29P*** confers partial protection against the reduction. Sham Veh n = 12, Sham ***29P*** n = 12, TAC Veh n = 13, TAC ***29P*** n = 15. E, F Two-way ANOVA with Tukey's multiple comparison test (*p < 0.05, **p < 0.01, ***p < 0.001, ****p < 0.0001). G Following TAC, expression of *Ace* and *Mme* were partially rescued by ***29P***. Sham Veh n = 5, Sham ***29P*** n = 3, TAC Veh n = 3, TAC ***29P*** n = 6, data from bulk RNA-sequencing of the left ventricle. Data analysed using DeSeq2, *p < 0.05, **p < 0.01, ***p < 0.001, ****p < 0.0001. (For interpretation of the references to colour in this figure legend, the reader is referred to the web version of this article.)

To determine the cell types in which αvβ3 integrin is expressed post-TAC, a search of the GEO repository was performed. Utilising data from Froese et al., 2022 (GSE180720 [32]), at 1-week post-TAC, the gene encoding β3 integrin, *Itgb3*, was upregulated in isolated endothelial cells, cardiomyocytes and fibroblasts (Fig. 1A). By 8-weeks post-TAC, *Itgb3* expression decreased to that of Sham control mice in endothelial cells and fibroblasts but remained significantly elevated in cardiomyocytes (Fig. 1A). These data indicate that within the heart, upregulated *Itgb3* expression is transient depending on the cell type.

Treatment with 50 μg/kg ***29P*** by oral gavage 1-week following surgery for 2 weeks had no significant effect on survival or body weight throughout the study (Fig. 1B and Supplementary Fig. 1A and B), suggesting no overt toxic effects, similar to Cilengitide. TAC initiates cardiac hypertrophy and chronic maladaptive remodelling can result in heart failure (HF). At the 3-week endpoint, increased heart weight to tibia length (Hw:Tl) and lung weight to tibia length (Lw:Tl) ratios were observed in Veh treated TAC mice when compared to its Sham control, indicative of cardiac hypertrophy and lung congestion, respectively (Fig. 1C). However, ***29P***-treatment partially prevented the increase in Hw:Tl ratio and lung congestion (Lw:Tl) was absent when compared to Veh TAC controls (Fig. 1C). We have previously shown that intervention at 3 weeks post-AAC with 3 weeks of ldCil, restores its cardioprotective effects at 6-weeks thus reversing maladaptive remodelling; effects which are sustained at 12-weeks [16]. As such, we sought to determine if our model of TAC and ***29P*** treatment could sustain its cardioprotective effects (Fig. 1B). However, at the 6-week experimental endpoint, *i.e.* 3 weeks following treatment cessation, ***29P*** treatment showed no difference in Hw:Tl or Lw:Tl (Supplementary Fig. 1C). Overall, these data suggest that immediately after treatment cessation, but not 6 weeks post-TAC *i.e.*, 3 weeks post-treatment cessation, ***29P*** confers protection against cardiac remodelling and prevents early signs of lung oedema, a phenotype associated with cardiac failure.

Progressive remodelling ultimately affects cardiac function. Although initially there may be some element of compensation, over time, decompensation results in loss of function which can be characterised *in vivo* using transthoracic echocardiography (Fig. 1D). Parameters obtained pre-treatment showed reduced cardiac function (assessed through fractional shortening [FS] and ejection fraction [EF]) was present following 1-week TAC (Fig. 1E). However, at the 3-week experimental timepoint, ***29P*** treatment of TAC mice significantly, albeit partially, prevented the deterioration of cardiac function when compared to Veh treated TAC controls (Fig. 1F). This effect was not sustained significantly at the 6-week experimental endpoint, 3 weeks following ***29P*** treatment cessation (Supplementary Fig. 1D).

Changes to kidney mass have been reported following TAC when the aorta is similarly constricted with a 27G needle [33]. In this study, following TAC, we observed a trend towards a reduced kidney mass and decreased kidney mass:TL ratio in the absence of any phenotypic differences (Supplementary Fig. 2A–D). At 3-week post-TAC, Veh treated TAC mice exhibit altered expression of key genes involved in the renin-angiotensin aldosterone system, namely *Ace* (angiotensin converting enzyme) and *Mme* (membrane metalloendopeptidase) and treatment with ***29P*** partially restored their expression levels towards those seen in Sham controls (Fig. 1G). However, at the 6-week experimental endpoint, the expression of *Ace* and *Mme* were comparable between Veh- and ***29P***-treated TAC mice (Supplementary Fig. 2E). These data show that ***29P*** treatment is unable to alleviate the loss of kidney mass following TAC.

### 3.2 *29P* alters genes associated with cardiac metabolism

Although ***29P*** has been successfully administered in mouse cancer models prior to this study [21], its potential mechanism of action(s) have not yet been explored in the heart. To investigate changes to gene expression following treatment with ***29P***, left ventricle apical tissue from all groups was subject to bulk RNA-Sequencing. According to the exclusion criteria detailed in the methodology, 2 libraries corresponding to one ***29P*** Sham and one Veh TAC were removed. Furthermore, following initial principal component analysis, a further Veh TAC mouse sample library was removed owing to a clustering association with Sham mice (data not shown). Removal of these three libraries followed by GSA analysis resulted in the identification of 13,800 genes. Hierarchical clustering and principal component analysis of the remaining 17 libraries revealed a clear clustering based on type of surgery and not to treatment type (Supplementary Fig. 3A and B). Changes to gene expression after TAC have been explored previously and more recently through interrogation of murine TAC with reference to human aortic stenosis datasets, all of which are available through the GEO repository [[34], [35], [36], [37]]. For this study, we focus on changes to gene expression caused by the administration of ***29P***. Regarding ***29P*** sham mice, RNA sequencing revealed 942 altered transcripts between Veh Sham and the ***29P*** treated Sham group (Supplementary Table 1). Gene ontology biological process (GOBP) enrichment analysis of these DEGs revealed enrichment of genes in processes involved in cytoplasmic translation, ribosomal small subunit assembly and various mitochondrial functions (data not shown). However, as we observed no differences to survival or the overall phenotype and cardiac function in ***29P*** Sham mice, the following analysis will focus on changes to gene expression in ***29P*** TAC mice.

**Fig. 2:**
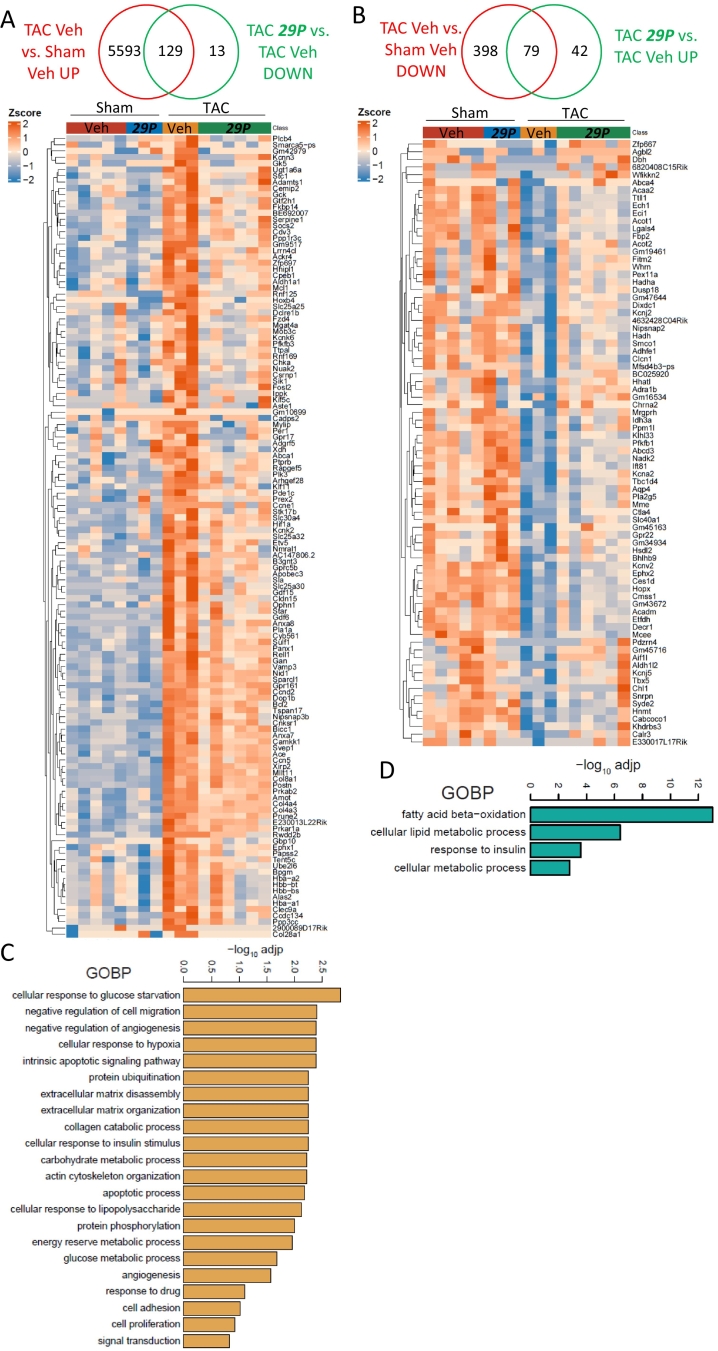
Analysis of upregulated and downregulated DEGs following TAC reveals ***29P*** may impact expression of genes involved in the response to hypoxia and angiogenesis Venn diagram illustrating downregulated (A) and upregulated (B) genes following ***29P*** treatment when compared to changes observed between Veh TAC and its respective Sham control. Overlapping genes are represented in the heat map with associated Z-score. Gene ontology enrichment analysis of these genes was performed and biological pathways of interest for each subset of DEGs is displayed as a box plot for downregulated (C) and upregulated (D) genes.

**Fig. 3:**
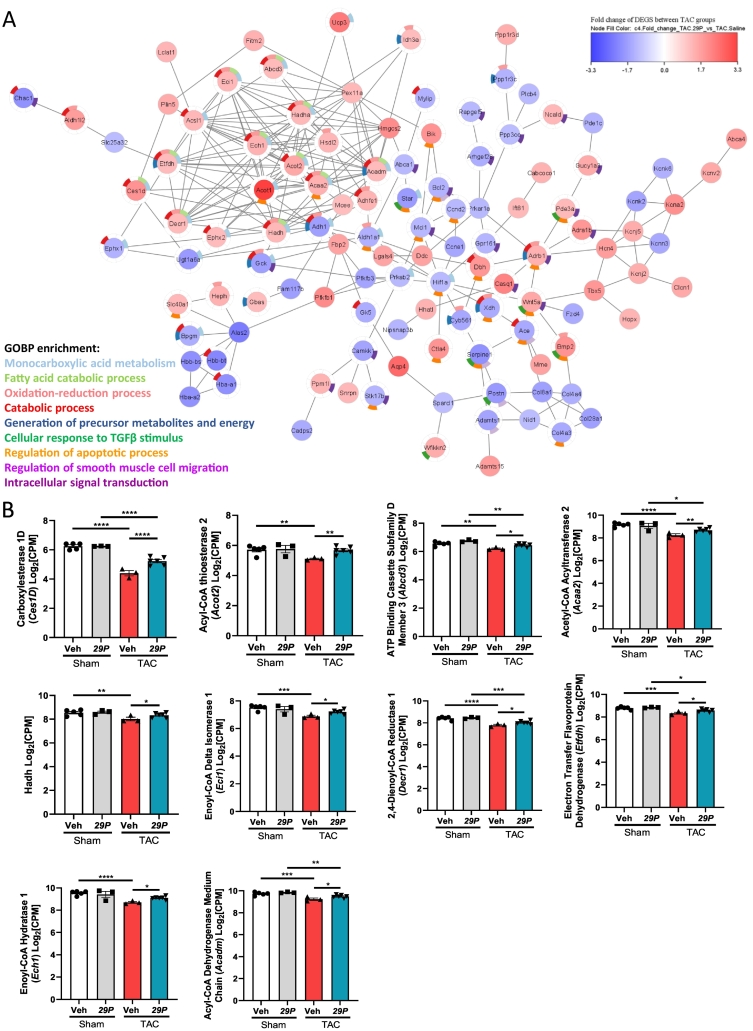
DEGs identified following ***29P*** treatment in TAC show enrichment in metabolic pathways. A Cytoscape STRING network analysis of DEGS between Veh- and ***29P***-treated TAC mice. Each node contains the gene name of one DEG with interacting partners indicated by connections. Fold change indicator: blue down- and red up-regulated transcripts. Colour points surrounding nodes show select GOBP enrichments. B All genes were significantly reduced in Veh TAC mice and treatment with ***29P*** restores transcript expression levels towards those observed in Sham controls. Gene panel: *Ces1d* (Carboxylesterase 1D), *Acot2* (Acyl-CoA thioesterase 2), *Abcd3* (ATP Binding Cassette Subfamily D Member 3), *Acaa2* (Acetyl-CoA Acyltransferase 2), *Hadh* (Hydroxyacyl-CoA Dehydrogenase), *Eci1* (Enoyl-CoA Delta Isomerase 1), *Decr1* (2,4-Dienoyl-CoA Reductase 1), *Etfdh* (Electron Transfer Flavoprotein Dehydrogenase), *Ech1* (Enoyl-CoA Hydratase 1) and *Acadm* (Acyl-CoA Dehydrogenase Medium Chain). Sham Veh n = 5, Sham ***29P*** n = 3, TAC Veh n = 3, TAC ***29P*** n = 6, data analysed using DeSeq2 (*p < 0.05, **p < 0.01, ***p < 0.001, ****p < 0.0001). (For interpretation of the references to colour in this figure legend, the reader is referred to the web version of this article.)

Firstly, we obtained DEGs between Veh TAC and Veh Sham and then compared this gene list with DEGs downregulated following ***29P*** treatment (Fig. 2A). This identified 129 genes of interest, GOBP enrichment analysis identified pathways key to pathological features associated with TAC such as ECM function(s), angiogenesis and various metabolic processes (Fig. 2C). We then performed the opposing analysis and identified downregulated DEGs between TAC Veh and Sham Veh and interrogated these against DEGs upregulated following ***29P*** treatment. From this analysis, 79 genes were identified (Fig. 2B). Of these genes, GOBP enrichment terms were related to fatty acid and metabolic processes and the response to insulin (Fig. 2D). To determine if the genes within the GOBP pathways interacted and to what extent, Cytoscape STRING network analysis of DEGs between Veh- and ***29P***-treated TAC mice was performed. STRING identified a large network with a subset of genes forming a large interacting subcluster encompassing both upregulated and downregulated transcripts (Fig. 3A). Analysis of the subcluster revealed an enrichment to pathways important in metabolic processes (Fig. 3A). TAC-induced changes to several metabolic genes were small but partially rescued with ***29P*** treatment (Fig. 3B).

### 3.3 Enrichment analysis of DEGs suggests a putative role for *29P* in the response to hypoxia and regulation of angiogenesis

**Fig. 4:**
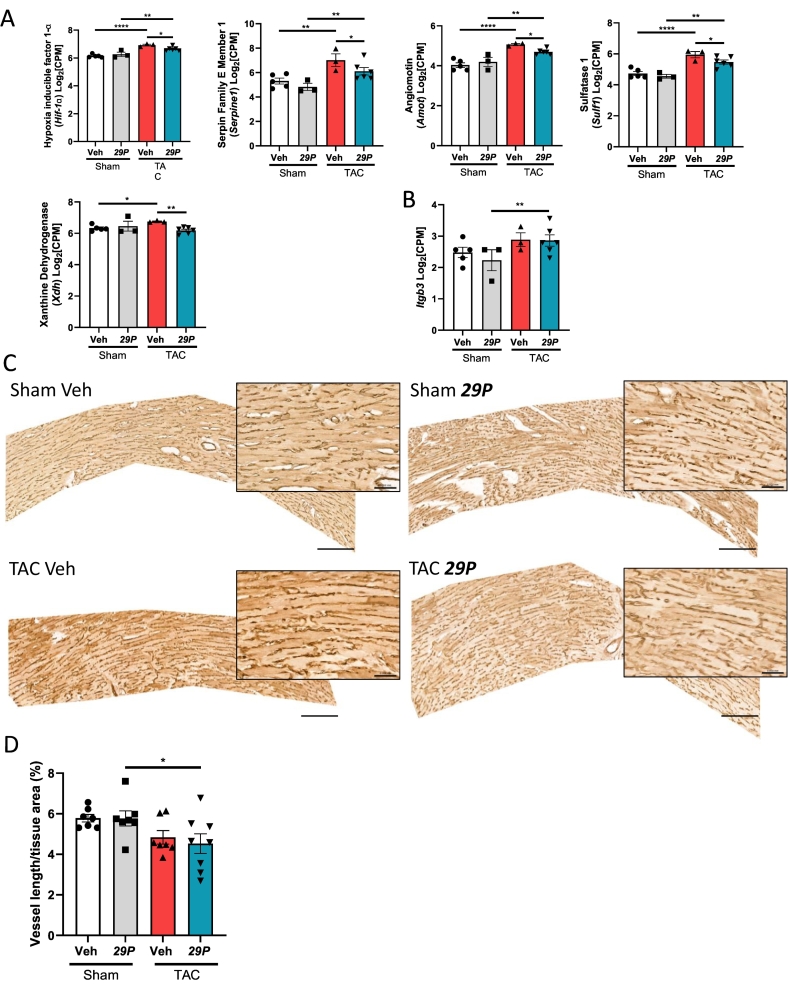
***29P*** treatment alters some angiogenic modulators but does not impact vessel area. A Expression of transcripts involved in hypoxia and angiogenesis which are differentially expressed between Veh- and ***29P***-treated TAC mice. Gene panel: *Hif1α* (Hypoxia inducible factor 1-α), *Serpine1* (Serpin Family E Member 1), *Amot* (Angiomotin), *Sulf1* (Sulfatase 1) and *Xdh* (Xanthine Dehydrogenase). B *Itgb3* is upregulated in ***29P*** treated TAC mice when compared to its respective Sham control. Sham Veh n = 5, Sham ***29P*** n = 3, TAC Veh n = 3, TAC ***29P*** n = 6, data analysed using DeSeq2 (*p < 0.05, **p < 0.01, ***p < 0.001, ****p < 0.0001). C Representative images of isolectin stained left ventricular myocardium at 3 weeks post-TAC cropped to longitudinal vessels used for analysis in (D). Scale bar = 200 μm. Inset scale bar = 50 μm. D Percentage of vessels per tissue area is reduced in ***29P*** treated TAC mice when compared to their respective sham controls. Sham Veh n = 7, Sham ***29P*** n = 7, TAC Veh n = 7, TAC ***29P*** n = 7, data analysed using two-way ANOVA with Tukey's post-hoc multiple comparison test (*p ≤ 0.05).

Changes to the cardiac microvasculature have been widely reported following TAC, with an initial reduction vessel density followed by a recovery period over 7–14 days [38]. We have reported previously that ldCil increased the density of blood vessels within the myocardium following AAC [16] and that ***29P*** increased tumour vasculature *in vivo* [21]. DEGs identified following RNA-sequencing, suggest that ***29P*** may alter genes involved in the regulation of angiogenesis and hypoxia (see Fig. 2C). Specifically, when compared with Veh TAC mice, ***29P***-treated TAC mice exhibit a reduction in the expression of *Hif1α* (Hypoxia inducible factor 1-α), a known regulator of hypoxia; *Serpine1* (Serpin Family E Member 1), a negative regulator of angiogenesis [39]; *Amot* (Angiomotin), which is known to have both pro- and anti-angiogenic effects [40]; *Sulf1* (Sulfatase 1), an inducer of postinfarct angiogenesis [41] and *Xdh* (Xanthine Dehydrogenase), an inhibitor of angiogenesis [42] (Fig. 4A). Furthermore, in ***29P***-treated TAC mice there was an increase in the expression of *Itgb3*, the gene encoding β3 integrin, when compared to its respective Sham control but no significant changes were found between Veh groups (Fig. 4B). However, at the 6-week timepoint there were no changes to *Hif1α* or *Itgb3* expression between groups (Supplementary Fig. 4A and B).

Owing to the proposed mode of action of ***29P*** and the DEGs identified in this study, we sought to determine if changes to the cardiac microvasculature were present in the heart following TAC (Fig. 4C). Comparing analysis of the vascular area at the 3-week timepoint between Veh treated Sham and Veh treated TAC showed that there was no significant loss of vessels in this model. ***29P*** had no apparent effect on vessel area after TAC when compared with Veh treated mice (Fig. 4D). At the 6-week timepoint *i.e.* 3 weeks post-treatment cessation, no change to vessel area was observed between any of the groups (Supplementary Fig. 4C).

### 3.4 *29P* treatment reduced left ventricular interstitial fibrosis and cardiomyocyte hypertrophy at 3 weeks post-TAC

**Fig. 5:**
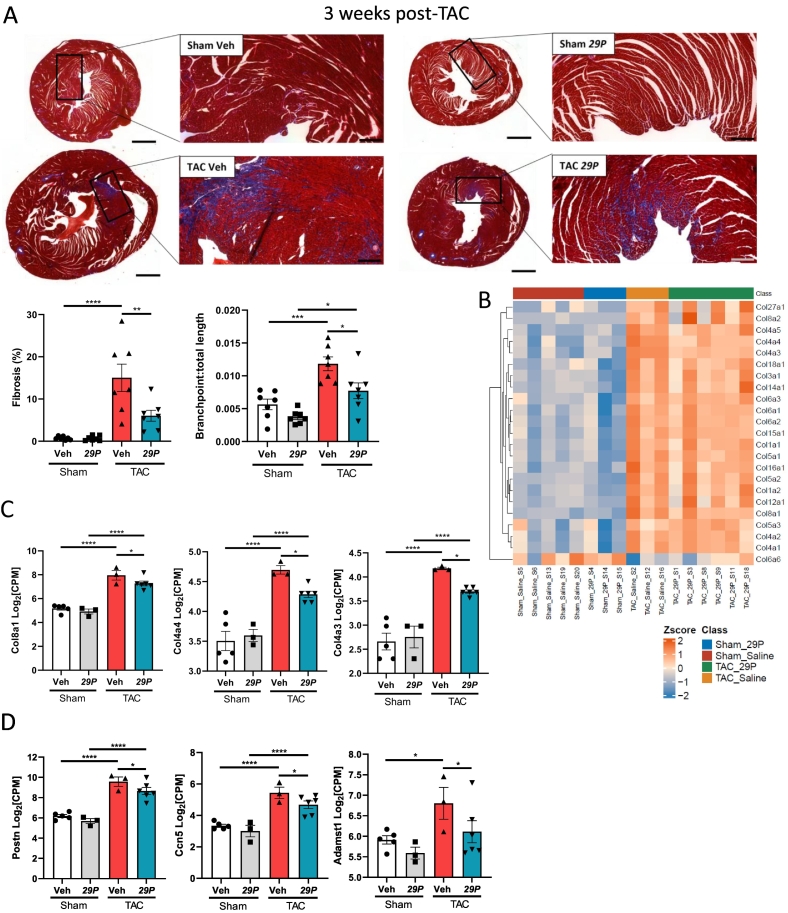
***29P*** treatment reduced interstitial fibrosis within the left ventricle at 3 weeks post-TAC. A Representative images of Masson's trichrome stained tissue and respective quantification of percentage left ventricular fibrosis and TWOMBLI fibre analysis. Scale bar(s): whole heart = 1 mm, inset = 200 μm. Quantitation of percentage LV fibrosis and branchpoint number increased after TAC and was significantly reduced by ***29P***. Sham Veh n = 7, Sham ***29P*** n = 7, TAC Veh n = 7, TAC ***29P*** n = 7, data analysed using two-way ANOVA with Tukey's post-hoc multiple comparison test (*p < 0.05, **p < 0.01, ***p < 0.001). B Hierarchical clustering heat map of collagen genes derived from MSigDB M3005 [44]. Expression levels of collagen genes (C) and collagen-associated genes (D) which are reduced in ***29P*** treated TAC mice compared to Veh-treated TAC mice. Data analysed using DeSeq2 (*p < 0.05, **p < 0.01, ****p < 0.0001). Sham Veh n = 5, Sham ***29P*** n = 3, TAC Veh n = 3, TAC ***29P*** n = 6.

The induction of pressure-overload causes reactive fibrosis, in which collagen is deposited in the absence of widespread cardiomyocyte death [43]. At 3 weeks post-TAC, both TAC groups showed an increase in the percentage of interstitial fibrosis and branchpoints when compared to their respective Sham controls. However, at 3 weeks post-TAC we observed reduced fibrosis and fewer branchpoints per total length of fibrosis in the ***29P*** TAC treatment group when compared to Veh TAC controls (Fig. 5A), suggesting ***29P*** treatment can reduce the occurrence of reactive fibrosis.

To determine if the reduction in fibrosis observed in ***29P***-treated TAC was associated with changes in genes associated with collagen formation, deposition and organisation we utilised MSigDB (v7.5.1, [M3005 [44]], [M23168], [M10505] and [M25228]). Following interrogation of these annotated pathways we identified several DEGs between TAC ***29P*** and TAC Veh important in collagen formation (MSigDB M3005 [44]). Both Veh-and ***29P***-treated TAC mice exhibited significant upregulation of a number of collagen genes, including the well-characterised genes *Col1a1* and *Col3a1* (Fig. 5B). Of these differentially expressed collagen genes, ***29P***-treatment induced a significant reduction in *Col8a1* (collagen Type VIII α-3), *Col4a3* (collagen Type IV α-3) and *Col4a4* (collagen Type IV α-4) when compared with Veh TAC controls (Fig. 5C). In addition, utilising RNA-seq, we identified a reduction in *Postn* (Periostin), *Ccn5* (cellular communication network 5) and *Adamts1* (ADAM metallopeptidase with thrombospondin type 1 motif 1) in ***29P*** treated TAC mice; these genes have also been implicated in collagen fibrillogenesis and ECM organisation (Fig. 5D). These data suggest that ***29P*** treatment can alter the expression of some key genes involved in the deposition of fibrosis and ECM reorganisation which are associated with a reduction in interstitial fibrosis.

**Fig. 6:**
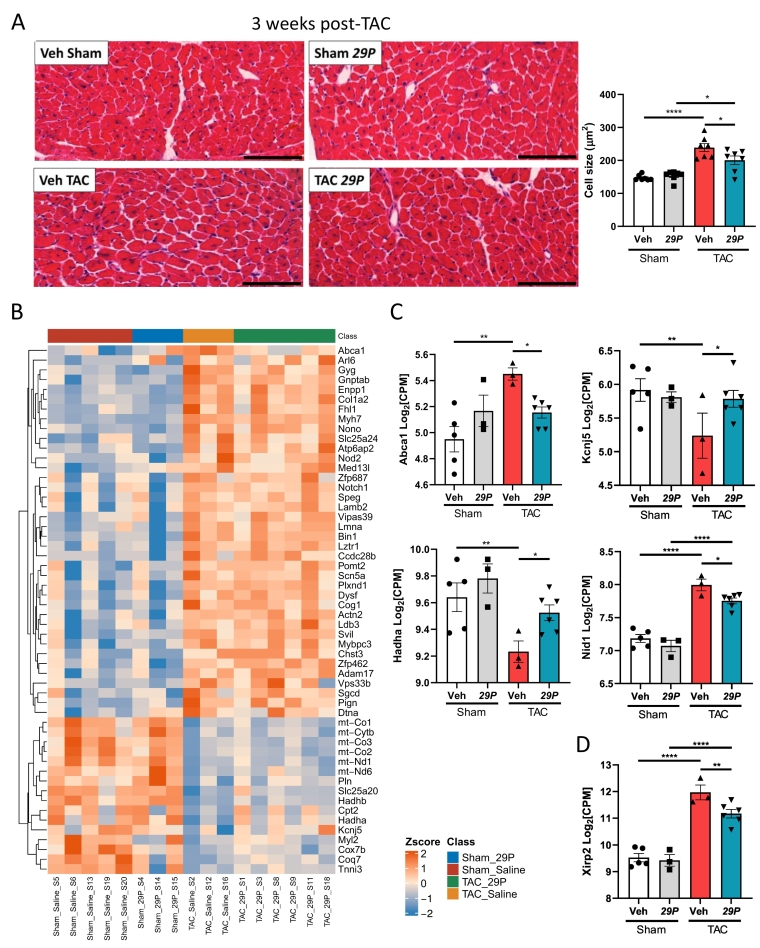
***29P*** partially rescues cardiac myocyte hypertrophy within the left ventricle at 3 weeks post-TAC and is associated with altered expression of hypertrophic genes. A Representative images from H&E stained sections and quantification of cardiomyocyte cross-sectional area showed the increased area following TAC is partially rescued by ***29P***. Scale bar = 100 μm. Sham Veh n = 7, Sham ***29P*** n = 7, TAC Veh n = 7, TAC ***29P*** n = 7, data analysed using two-way ANOVA with Tukey's post-hoc multiple comparison test (*p < 0.05, ****p < 0.0001). B Hierarchical clustering heat map of MSigDB (M35413) annotated genes involved in human LV hypertrophy. C DEGs altered after TAC and their abundance significantly rescued by *29P* treatment include: *Abca1* (ATP-binding cassette sub-family A member 1), *Kcnj5* (Potassium Inwardly Rectifying Channel Subfamily J Member 5) and *Hadha* (hydroxyacyl-CoA dehydrogenase trifunctional multienzyme complex subunit-α). D Expression of additional pro-hypertrophic genes altered in *29P* treated TAC (MSignDB [M14043 [45]]). Data analysed using DeSeq2 (*p < 0.05, **p < 0.01, ****p < 0.0001). Sham Veh n = 5, Sham *29P* n = 3, TAC Veh n = 3, TAC *29P* n = 6.

Another hallmark of pressure-overload induced cardiac hypertrophy is the concomitant increase in cardiomyocyte cross-sectional area (CSA). At 3 weeks post-TAC, cardiomyocyte CSA increases in both Veh- and ***29P***-treated TAC mice when compared with their respective Sham controls (Fig. 6A). Interestingly, ***29P*** treatment partially rescued the increase in cardiomyocyte CSA when compared to Veh-treated TAC mice (Fig. 6A). To determine if changes to genes associated with cardiac hypertrophy were observed following ***29P*** treatment, we again interrogated DEGs identified in our RNA-seq database using annotated human hypertrophic pathways derived from MSigDB (v7.5.1 [M35413] and [M14043 [45]]) (Fig. 6B). Genes in these pathways were explored due to their annotation with pathological changes including an increase width of the LV wall with loss of elasticity and enlargement of LV mass. ***29P*** treatment rescued the expression of some genes from these annotated pathways and thus these data suggest that ***29P*** may be involved in the regulation of these processes (Fig. 6C). Furthermore, TAC resulted in an increased expression of additional well-characterised pro-hypertrophic genes such as *Nppa*, *Nppb* (data not shown) and *Xirp2* (Xin Actin Binding Repeat Containing 2). Treatment with ***29P*** partially restored the expression level of *Xirp2* towards those observed in Sham controls (Fig. 6D).

### 3.5 Cardioprotective properties conferred by *29P* treatment are lost at 6 weeks post-TAC *i.e.* 3 weeks following treatment cessation

As discussed briefly above, 3 weeks following treatment cessation (*i.e.* at 6 weeks post-TAC) functional data derived from echocardiography showed that there was no difference between TAC groups, suggesting that the cardioprotective functional effects conferred by ***29P*** at 3-weeks post-TAC are lost following treatment withdrawal. At the 3-week timepoint we identified structural changes in TAC mice treated with ***29P***; however, 6-weeks post-TAC, there was no difference to percentage fibrosis in the left ventricle, TWOMBLI-assessed number of fibrosis branchpoints (Supplementary Fig. 5A and B) or *Col4a3* and *Col4a4* transcripts between Veh- and ***29P***-treated TAC mice (Supplementary Fig. 5C). We then evaluated cardiomyocyte CSA and observed an increased cell area after TAC but no difference between Veh and ***29P*** treatment groups (Supplementary Fig. 5D). Overall, the beneficial effects of ***29P*** treatment are lost 3 weeks post-treatment cessation.

## 4 Discussion

Despite advances to our knowledge and understanding of HF, there remains an unmet clinical need for novel therapeutics which can both prevent and reverse established cardiac remodelling with minimal to no side effects. Integrins are well characterised interactors of the extracellular matrix, making them therapeutically targetable [46]. Previous studies have implicated β3 integrin and its downstream signalling as a disease-modifying mediator of cardiac remodelling [47,48]. These studies established a role for low doses of the RGD-mimetic, αvβ3/αvβ5 integrin-specific, cyclic peptide Cilengitide in preventing adverse cardiac remodelling and increasing BV density in an AAC model of pressure-overload cardiac hypertrophy [16]. Given these promising results *in vivo*, we sought to determine if the second generation orally available RGD-mimetic prodrug ***29P***, which we have shown harbours a pro-VEGF stimulated angiogenic effect in subcutaneous tumours and in *ex vivo* aortic ring sprouting assays [21], would confer similar cardioprotective properties to ldCil in a TAC model of pressure-overload induced cardiac hypertrophy and HF.

The data in this study demonstrate the cardioprotective capability of ***29P***. Specifically, at 3 weeks post-TAC following 2 weeks of treatment, ***29P*** can improve cardiac function, attenuate cardiomyocyte hypertrophy and reduce interstitial fibrosis. The ability of ***29P*** to confer these different cardioprotective affects correlate with increased β3 integrin transcript expression after TAC. *Itgb3* is detectable in cardiac endothelial cells, cardiomyocytes and cardiac fibroblasts and its expression is elevated in all these cell types at 1-week post-TAC [32], suggesting *Itgb3* may play a potential role in angiogenesis, hypertrophy and reactive fibrosis in the acute phase of pressure overload, respectively.

ldCil treatment was shown to alter the profile of putative and known cardioprotective regulators [16]. Similarly, here, apical left ventricle RNA-seq at 3 weeks post-TAC reveals changes to the expression profile of known and putative genes involved in cardiac metabolism, hypertrophy, deposition of the basement membrane and angiogenesis. Treatment with ***29P*** restores key transcriptomic profiles towards levels observed in the Sham controls. Direct involvement between the metabolism genes identified in this study and αvβ3 has not been well documented. However, in glioblastoma cells, αvβ3 interaction with the ECM protein osteopontin promotes aerobic glycolysis [49], and β3 knockout mice have a higher serum triglyceride level caused by disrupted lipoprotein lipase secretion [50]. Therefore, αvβ3 in other models can help regulate aspects of metabolism but in the heart a function for αvβ3 has not been elucidated.

In this study, ***29P*** significantly reduces the deposition of collagen fibres and cardiomyocyte hypertrophy throughout the LV. In accordance with this, expression of collagen genes such as *Col4a3* and *Col4a4* as well as the gene encoding the ECM protein NID1 are all downregulated. NID1 is an important component of the vascular basement membrane and acts as a linker protein of COL4 and laminin [51]. Recent *in vitro* studies have implicated NID1 in the transdifferentiation of fibroblasts into ECM depositing myofibroblasts [52] and in the activation of the mitogen activated protein kinase pathway in cardiomyocytes through αvβ3 binding [53]. This potential role of NID1 signalling offers a potential mechanism of action of ***29P*** treatment in non-endothelial cells. RNA-seq also revealed a reduction in the cardiac stress marker *Xirp2* in ***29P*** TAC mice. *Xirp2* has been identified through single cell sequencing to be upregulated in sub-clusters of cardiomyocytes from human hypertrophic cardiomyopathy cardiac samples [54] and mutations within *Xirp2* are linked to dilated cardiomyopathy [55] highlighting a further potential cardioprotective mechanism of ***29P***.

There is a growing body of evidence that cardiac microvasculature rarefaction in the failing heart leads to worsening cardiac perfusion and poor prognosis [56], indeed HF onset can be delayed in preclinical models when angiogenesis is stimulated [57]. We have shown that ***29P*** increases tumour microvasculature *in vivo* [21], despite this we fail to observe a change to microvascular area in the heart at 3 weeks post-TAC, following 2 weeks of ***29P*** treatment. This is also in contrast to our previous study in which ldCil treatment after AAC induced an increase in the cardiac microvasculature [16]; this disparity could be due to a number of reasons: 1) the model of HF utilised differs *i.e.* TAC *vs* AAC, 2) the time points at which treatment was commenced and its duration, 3) the experimental time point at which BV were assessed, 4) the anti-fibrotic and anti-hypertrophic effect of ***29P*** reduces the overall hypoxic environment in the LV myocardium. In keeping with the latter point, *Hif1α* levels are reduced in ***29P***-treated TAC mice when compared to Veh TAC controls.

In this present study, ***29P*** was cardioprotective at 3 weeks post-TAC, following 2 weeks of treatment. Whilst the features described in detail above indicate that ***29P*** can help prevent the typical progression of TAC, they do not address whether the cardioprotective benefits were a permanent feature or reversible. To investigate this, we withdrew treatment at 3 weeks post-TAC and continued to monitor TAC progression through to 6 weeks. Here, we showed that when ***29P*** treatment is halted, its cardioproctective effects are lost. The apparent loss of cardioprotection could be due to the simple fact that the aorta remains banded, therefore the driving force behind cardiac decompensation *i.e.* pressure-overload, is still present. As such, we could speculate that ***29P*** confers its affects in a temporal, reversible manner and sustained administration of ***29P*** would be required for prolonged cardioprotective effects.

Compound ***29P*** has been engineered to be orally available and binds more with more affinity to αvβ3 over any other integrin [21] thus overcoming one of the key challenges of peptide/antibody promiscuity in integrin targeting. The first generation RGD mimetic pentapeptide Cilengitide has been administered in around 30 clinical trials for cancer and, although largely well-tolerated, does have some known adverse events (haematological, neuropathological, liver toxicity) [58]. In this study, treatment with ***29P*** did not result in any notable adverse events in either control (sham) or TAC treated groups. Control sham mice were subject to the same ***29P*** treatment and importantly, did not display any changes to cardiac structure, function or overall survival. However, whole RNA sequencing did reveal changes to transcripts under these baseline conditions which could warrant further investigation under long-term conditions to ensure these changes do not lead to any adverse events. αvβ3 integrin has also been targeted by a variety of other therapeutics with some progressing to clinical trials. These include the small molecules SFO166, Risuteganib and SB-273005 for the treatment of retinopathies and osteoporosis and a humanised monoclonal antibody, VPI-2690B, for the treatment of diabetic nephropathy [59]. Of the aforementioned αvβ3-targeting therapies only the monoclonal antibody is specific for αvβ3; however, all were reported to be safe, further suggesting that ***29P*** would be well tolerated. ***29P*** has been previously utilised by our group in a murine model of Lewis Lung carcinoma and was well-tolerated with no observed side-effects or off-target effects when administered either orally or given in its protracted ***29*** form [21].

Overall, our results suggest that utilising ***29P*** for the treatment of pathological hypertrophic remodelling has potential and warrants further long-term investigation. Although beyond the scope of this present study, given that ***29P*** can reduced fibrosis and improve cardiac function, it would be interesting to explore the use of ***29P*** for treatment of post-myocardial infarction.

### 4.1 Limitations

One of the main aims of this study was to determine the use of the orally available prodrug ***29P*** over several weeks after TAC surgery. However repeated oral gavage in mice can cause adverse effects over several weeks that could affect the wellbeing of the animals. Thus sustained long-term studies would likely require the use of a mini-pump device to either administer the cleaved product ***29*** subcutaneously or ***29P*** into the stomach through a catheter attached to the mini-pump. However, these also have a limited capacity of maximum 6 weeks before requiring further surgery for replacement. The study has been limited to 3- and 6-week time points. Future studies would determine effects between these time points in order to pinpoint when the beneficial effects of ***29P*** treatment stop after treatment cessation.

## Funding

AN 10.13039/501100000274British Heart Foundation (PG/18/75/34096). LL and EJC 10.13039/501100000274British Heart Foundation (FS/18/62/34183). AC and EJC 10.13039/501100000274British Heart Foundation (FS/4yPhD/20/34131). NS 10.13039/501100000274British Heart Foundation (RG/F/21/110055). JH, MZ and SP are employed by the University of Manchester. CG, AH, MW and HK did not receive any specific grant from funding agencies in the public, commercial, or not-for-profit sectors. EM and JW acknowledge the support from the 10.13039/501100000289CRUK City of London Major Centre core funding to Barts Cancer Institute. JW is HEFCE funded by 10.13039/100009148Queen Mary University of London. KHD is employed by Queen Mary University of London and is HEFCE funded by 10.13039/100009148Queen Mary University of London, Barts Cancer Institute, 10.13039/501100000289CRUK City of London Major Centre; LW was employed at Queen Mary University London, Barts Cancer Institute and supported by the 10.13039/501100000289CRUK City of London Major Centre.

## CRediT authorship contribution statement

**Alexandra Njegić:** Data curation, Formal analysis, Investigation, Methodology, Writing – original draft, Writing – review & editing. **Lina Laid:** Data curation, Methodology, Writing – review & editing. **Min Zi:** Data curation, Methodology, Writing – review & editing. **Eleni Maniati:** Data curation, Formal analysis, Methodology, Writing – review & editing. **Jun Wang:** Supervision. **Alexandru Chelu:** Data curation, Formal analysis, Methodology, Writing – review & editing. **Laura Wisniewski:** Formal analysis, Methodology, Writing – review & editing. **Jenna Hunter:** Methodology. **Sukhpal Prehar:** Data curation, Methodology. **Nicholas Stafford:** Data curation, Writing – review & editing. **Chaim Gilon:** Resources, Writing – review & editing. **Amnon Hoffman:** Resources, Writing – review & editing. **Michael Weinmüller:** Resources, Writing – review & editing. **Horst Kessler:** Resources, Writing – review & editing. **Elizabeth J. Cartwright:** Supervision. **Kairbaan Hodivala-Dilke:** Conceptualization, Funding acquisition, Supervision, Writing – original draft, Writing – review & editing.

## Declaration of competing interest

The authors declare the following financial interests/personal relationships which may be considered as potential competing interests: Kairbaan Hodivala-Dilke reports a relationship with Ellipses Pharma that includes: consulting or advisory. Kairbaan Hodivala-Dilke reports a relationship with Vasodynamics that includes: consulting or advisory. Kairbaan Hodivala-Dilke reports a relationship with RGDscience Ltd that includes: consulting or advisory. Horst Kessler and Amnon Hoffman and Michael Weinmuller has patent #WO2019058374A1 pending to YISSUM RES DEV CO OF HEBREW UNIV JERUSALEM LTD [IL]; UNIV MUENCHEN TECH [DE]. Kairbaan Hodivala-Dilke has patent #WO2021032955A1 pending to UNIV LONDON QUEEN MARY [GB]. If there are other authors, they declare that they have no known competing financial interests or personal relationships that could have appeared to influence the work reported in this paper.

## Data Availability

Data generated through RNA-sequencing of the left ventricle apical tissue at 3 weeks post-TAC is available at the GEO repository, GSE247309.

## Appendix A Supplementary data

Supplementary material Image 1
