# Supplementary material for: Treatment with αvβ3-integrin-specific 29P attenuates pressure-overload induced cardiac remodelling after transverse aortic constriction in mice
Source: J Mol Cell Cardiol Plus. 2024 Mar 12;8:100069. doi: 10.1016/j.jmccpl.2024.100069 (PMC11196926; doi:10.1016/j.jmccpl.2024.100069)
Supplement: Supplementary file 1 — Supplementary material [file mmc1.docx]

# Supplementary Materials and Methods

## Echocardiography measurements

Utilising the M-mode image, the width of the interventricular septum (IVS), the internal diameter of the left ventricle (LVID) and the posterior wall (PW) in diastole (;d) and systole (;s) was measured (VisualSonics, FujiFilm). Cardiac function was estimated using calculations of fractional shortening {[(LVID;d-LVID;s)/LVID;d] x100} and cardiac ejection fraction {[(1.047xLVID;d)^3^-(1.047xLVID;s)^3^]/[(1.047xLVID;d)^3^] x 100} where 1.047 is myocardial specific gravity (g/ml).

## Histology

The mid-section of the heart was placed in 4% paraformaldehyde in PBS for 24 hours, following which they were transferred to 70% ethanol until tissue processing. All hearts were processed using an automated tissue processor (Leica). Hearts were embedded in paraffin wax and 5μm thick sections were obtained on poly-L-lysine coated slides using standard microtomy.

## Immunohistochemistry - automated

The protocols below were utilised when using the automated slide stainer (Leica). Prior to each staining, sections were de-paraffinised, placed in xylene for 10 minutes, and rehydrated in decreasing alcohol concentrations (100%, 90% and 75%).

### Haematoxylin and Eosin

Slides were rehydrated and incubated in Weigerts Iron Haematoxylin for 2 minutes, followed by washing in water. Slides were then placed in 5% acetic acid for 10 seconds, followed by washing in water and incubation in bluing reagent for 30 seconds. Slides were then washed in water, placed in 100% ethanol for 1 minute, eosin for 1 minute 30 seconds and then dehydrated, cleared and mounted.

### Masson’s trichrome

Following rehydration of slides, slides were placed in saturated picric acid for 60 minutes and washed in water for 7 minutes, followed by a 10-minute incubation in Weigerts Iron Haematoxylin. Slides were then placed in water for 10 minutes followed by a 12-minute incubation in Biebrich Scarlet Acid Fuchsin. Slides were then washed in water and placed in phosphomolybdic-phosphotungstic acid for 12 minutes, following a brief wash in water, slides were incubated in aniline blue solution for 7 minutes. Slides were again washed in water and then placed in 1% acetic acid for 2 minutes, followed by another 2-minute wash in water. Slides were then dehydrated in ethanol (95%, 100%) and cleared in xylene before mounting.

## Immunohistochemistry - manual

### Isolectin β4 staining

Briefly, the wax was melted by gentle heating (60^°^C) and slides were rehydrated through decreasing exposure to ethanol. Rehydrated slides were incubated with hydrogen peroxide block for 10 minutes (Abcam) followed by washing in Tris buffered saline containing 0.025% Triton X-100 (TBS-T). Sodium citrate (pH6)-mediated antigen retrieval was then performed by placing slides in a microwave pressure cooker and heating on high for 15 minutes. Sections were then blocked with avidin and biotin blocking reagents according to manufacturer’s instruction (Jackson). Slides were then washed in TBS-T (5 minutes, three times) and incubated with biotinylated isolectin β4 (Vector) diluted 1:100 in TBS-T. The following day, slides were washed in TBS-T (5 minutes, four times) and incubated with streptavidin peroxidase (Abcam) for 30 minutes at room temperature. Slides were then washed in TBS-T (5 minutes, four times) and sections were developed using the DAB chromogen/DAB substrate method (Abcam). After staining had developed, slides were washed in TBS-T (5 minutes, four times), counterstained with haematoxylin for 5 minutes, washed in warm water, dehydrated, cleared and mounted.

## Histological Quantification

### Left ventricle fibrosis

Individual Masson’s stained fibres were analysed using The Workflow Of Matrix BioLogy Informatics[1] (TWOMBLI) plugin according to the following parameters: Contrast Saturation 0.35, Line Width 5, Minimum Curvature Window 30, Maximum Curvature Window 90, Minimum Branch Length 5, Maximum Display High Density Matrix, 220. To calculate the parameters of individual fibres, 6-10 10x magnified images of each Masson’s Trichrome stained section were first deconvoluted using either the Masson’s Trichrome or H-Pas filter available on ImageJ. Following colour deconvolution, the image representing the blue fibrotic stain of Masson’s Trichrome was saved and then processed according the TWOMBLI pipeline and parameters detailed above. Calculation of normalised fibre values was performed by calculating the total value for fibre branchpoints and dividing this by the sum of the total fibre length[1].

### Blood vessel analysis

Briefly, 3 images acquired at 10x throughout the left ventricle were cropped to longitudinal vessels and then processed using an ImageJ Macro. Briefly, this macro utilised the following tools in sequence: convert to 8-bit, threshold (otsu, user to select manual threshold) to select the vessels, despeckle, Skeletonize (2D/3D) and finally Analyse Skeleton (2D/3D)[2]. All vessel lengths were included in the analysis; total vessel length was obtained by calculating the sum of all individual branch lengths.

## RNA extraction

After rough sectioning of excised hearts, the left ventricle apex was snap frozen in RNAlater (ThermoFisher) and stored at -80^o^C until processing. RNA was extracted from hearts using the TRIzol (ThermoFisher)/chloroform method. Briefly, hearts were defrosted, minced with a sterile scalpel, and then further homogenised in 500μl TRIzol using a Dounce homogeniser. Homogenates were then incubated at room temperature for 15 minutes, following which 100μl of chloroform was added and samples were incubated, inverted and then microcentrifuged (8,000g 15 minutes, 4℃). The upper aqueous layer was transferred to a clean Eppendorf and an equal volume of isopropanol added, samples were then inverted, incubated at RT for 10 minutes and microcentrifuged (10,000g, 10 minutes, 4℃). The supernatant was discarded, and the resultant pellet was washed in 500μl 75% ethanol in nuclease free water. Samples were microcentrifuged (8,000g, 10 minutes, 4℃) and the supernatant discarded, the resultant pellet was air dried and then resuspended in nuclease free water.

## Next generation RNA sequencing Illumina

### Preparation of mRNA library from total RNA

RNA samples were diluted to 20-25ng/μl using RNase-free water. 100ng of each sample and Ambion Human Brain Total RNA control was aliquoted to a final volume of 50μl in RNAse-free water. The first Strand Synthesis Buffer mastermix was prepared, vortexed and left on ice. To isolate, fragment and prime the mRNA 20μl of NEBNext Oligo d(T) beads per sample were placed into the well of PCR plate and were washed with 100μl of RNA Binding Buffer. The plate was then placed on a magnetic rack at RT for 2 minutes and the supernatant removed and discarded. This process was repeated a further 2 times. The beads were then re-suspended in 50μl of RNA Binding Buffer and the diluted RNA samples were added. The plate was sealed and placed in a thermocycler under the following conditions: 65^o^C for 5 minutes and then 4^o^C hold, lid temperature set at 70℃. The plate was then microcentrifuged and the beads re-suspended. The plate was incubated at RT for 5 minutes to enable the mRNA to bind to the beads. The plate was then placed on a magnetic rack and the supernatant removed and discarded. 200μl of wash buffer was added to each well and the plate was placed back onto the magnetic rack; the resultant supernatant was removed and discarded. This step was repeated a further time. 50μl Tris Buffer was then added to each well and the plate was placed in a thermocycler under the following conditions: 80℃ for 2 minutes and then 25℃ hold, lid temperature set at 90℃. The plate was then microcentrifuged and 50μl of RNA binding buffer was added to allow the mRNA to re-bind to the beads. The plate was incubated at RT for 5 minutes and then placed on a magnetic rack. The supernatant was removed, and the beads were washed with 200μl wash buffer, placed back onto the magnetic rack and the wash buffer supernatant was fully removed. mRNA was then eluted by adding 11.5μl of the First strand synthesis buffer mix to each well and incubating the plate in a thermocycler using the following conditions: 94℃ for 10 minutes. The plate was then placed on a magnetic rack and 10μl purified mRNA was collected. To synthesise cDNA, a First Strand Enzyme mastermix was made comprised of NEBNext Strand Specificity Reagent and NEBNext Frist Strand Synthesis Enzyme Mix, of which 10μl was added to each primed mRNA sample. The samples were placed in a thermocycler according to the following conditions: 25℃ for 10 minutes, 42℃ for 50 minutes, 70℃ for 15 minutes and then 4℃ hold, lid temperature set at 80℃. Following this, 60μl of Second Strand Mastermix was added to each sample (comprised of nuclease free water, NEBNext Second Strand Synthesis buffer and NEBNext Second Strand Synthesis Enzyme mix) and the samples were placed in a thermocycler according to the following conditions: 16℃ for 60 minutes. Double-stranded cDNA was then purified using 1.8X Agencourt AMPure XP beads; briefly, 144μl of resuspended AMPure XP beads were added to each sample, the samples vortexed and allowed to stand at RT for 5 minutes. The plate containing the samples was then placed on a magnetic rack and the supernatant removed and discarded. 200μl of 80% ethanol (in nuclease free water) was then added to the samples whilst on the magnetic rack. Following a 30 second incubation, the ethanol was removed. This process was repeated. After successive ethanol washes, the beads were air dried whilst on the magnetic rack. To elute the DNA, the plate was removed from the rack and 53μl 0.1X TE Buffer was added and the samples vortexed. The samples were incubated at RT for 2 minutes and then placed back onto the magnetic rack at which point 50μl supernatant was removed and stored at -20℃ until further processing.

The End Prep of the cDNA library was then prepared by adding 10μl of End Prep Mastermix (NEBNext Ultra II End Prep Reaction buffer and NEBNext Ultra II End Prep Enzyme Mix) to each cDNA sample. The samples were centrifuged and placed in a thermocycler under the following conditions: 20℃ for 30 minutes, 65℃ for 30 minutes, and then 4℃ hold, lid temperature set at 75^o^C. Following this, 2.5μl of diluted NEBNext Adaptor (1:25 in Adaptor dilution buffer), 30μl of the NEBNext Ultra II Ligation Master Mix and 1μl of NEBNext Ligation enhancer was added to each sample and the samples placed into a thermocycler under the following conditions: 20℃ for 15 minutes. 3μl USER Enzyme was then added and the samples place back unto a thermocylcer set at the following conditions: 20℃ for 15 minutes, lid temperature ≥45℃. The ligation reaction was then purified by adding 25μl of AMPureXP beads to each sample, incubating at RT for 5 minutes following by magnetic bead separation. All sample supernatant was transferred to a new plate and the beads discarded. 10μl of AMPureXP beads were then incubated with the supernatant at RT for 5 minutes, following which the plate was placed on a magnetic rack and the supernatant removed and discarded. The resultant beads were washed in 80% ethanol twice, each time the supernatant was discarded. The beads were left to air dry for 5 minutes. To elute the DNA, 17μl of 0.1X TE buffer was then added, the plate centrifuged and placed back on to a magnetic rack. 15μl of supernatant was then transferred to a clean PCR plate.

PCR library enrichment was performed by adding 25μl of NEBNext Q5 Hot Start HiFi PCR mastermix and 10μl of NEBNext Miltoplex Oligos for Illumina to each well and then placing the plate into a thermocycler programmed to the following: step 1. 30 seconds 98℃, step 2. 10 seconds 98℃ and 75 seconds 65℃ (13 cycles), step 3. 5 minutes at 65℃, step 4. 4℃ forever. The PCR reaction was then purified by adding 45μl Agencourt AMPure XP beads to each reaction sample, followed by 5 minutes RT incubation and removal of supernatant using a magnetic rack. The beads were then washed in 200μl ethanol twice, with a 30 second incubation period during each wash step. The beads were then air dried for 5 minutes. DNA was eluted by adding 23μl 0.1X TE buffer, followed by a 2-minute incubation at RT and placement onto a magnetic rack. After separation, 20μl of supernatant was removed and stored at -20℃.

### Quantification of mRNA libraries and fragment size quality control

Qubit standards and samples were prepared in Qubit working solution (1:200, Qubit Fluorescent Reagent:Qubit Dilution Buffer). Standards were first measured, followed by each sample. Following quantification of mRNA libraries, the fragment size of each library was calculated using an Agilent 4200 Tapestation (Agilent) using the HS D1000 and HS D5000 reagent kits. Briefly, 1μl D1000 and 2μl HS 5000 ladder were aliquoted into a 96-well plate and the samples prepared by adding 3μl D1000 and 2μl HS 5000 buffer to 1μl (for D1000) and 2μl (for HS 5000) of each sample, respectively. The plate was sealed with adhesive foil and inserted into an IKA M53 vortex for 1 minute. The plate was then pulse centrifuged and loaded into the Agilent 4200 Tapestation (Agilent). New screentape was placed into the Tapestation and the samples were run. This identified 4 libraries with mRNA fragments around 350bp in size and were subsequently pooled according to their individual molarities.

### Library dilution and pooling of normalised library dilutions

All stock libraries were diluted to a final working dilution of 3nM in 20μl. Libraries with a molarity of <3nM were pooled to a final working dilution of 2nM. After loading all diluted libraries, the plate was sealed with an adhesive PCR film, vortexed and microcentrifuged. The libraries were then cleaned to remove fragments <130bp such as unwanted primers. All samples were diluted in EB buffer to a volume of 50μl and 45μl of AMPure XP beads. The sample and bead solution was incubated at RT for 10 minutes and then placed on a magnetic rack. Beads were allowed to settle and the supernatant removed and discarded. The beads were washed with 80% ethanol twice (200μl ethanol, 30 second incubation, removal of supernatant) and then the beads were air dried for 10 minutes. The beads were removed from the magnet and resuspended in 40μl EB buffer. Samples were incubated at RT for 10 minutes, placed back on the magnet and the supernatant transferred to a Lo-bind 1.5ml tube. The cleaned mRNA libraries were then quantified using the Qubit 2.0 Fluorometer according to the protocol detailed above (quantification of mRNA libraries and fragment size quality control). mRNA library fragment size was then checked using the Agilent 4200 Tapestation (Agilent) using the HS D1000 reagent kit only according to the protocol detailed above (quantification of mRNA libraries and fragment size quality control).

### Illumina NextSeq library denaturation and loading

An equal volume of 0.2N NaOH was added to the library in a 1.5ml LoBind microcentrifuge tube, the library was vortexed and incubated at RT for 5 minutes. An equal volume of 200mM Tris-HCl was added and the library vortexed. HT1 buffer was then added to bring the volume to 1ml. The volume of denatured library equating to 1.2pM was then diluted in HT1 to a final volume of 1,300μl. 1.2μl of 20pM PhiX was then added to the library (the same volume of denatured library was removed to ensure the final loading volume of 1,300μl). The full volume of library was loaded into the sequencing cartridge and the flow cell, buffer cartridge and reagent cartridge were placed into their respective compartments and the sequencing run started.

### Supplementary analysis

Hierarchical clustering was performed using Log_2_[CPM] expression of all genes and the ward-D clustering method on a Pearson’s correlation matrix.

## Real time quantitative PCR

### DNase treatment and RNA-cDNA conversion

RNA quality and quantity was determined using a nanodrop (ND-800). 1μg of LV RNA and 250ng of iPSC-CM RNA was subject to DNAase treatment according to manufacturer’s guidance (Sigma), and then converted to cDNA using the high-capacity RNA-to-cDNA kit (Applied Biosystems). To remove any residual DNA fragments, amplification grade DNase1 and DNase1 reaction buffer was incubated with 1μg of RNA for 15 minutes at RT following which, samples were treated with 25nM EDTA stop solution heated to 70℃ for 10 minutes. Following which samples were incubated with 10x RT buffer, 25x dNTP mix, 10x ST random primers, multiscribe reverse transcriptase and nuclease free water and placed in a thermocycler programmed to the following: step 1. 10 minutes at 25℃, step 2. 120 minutes at 37℃, step 3. 5 minutes at 85℃, step 4. 4℃ forever.

### RT-qPCR Taqman protocols

Gene expression was determined using quantitative real time qPCR with Taqman (Applied Biosystems) reagents as follows: 1μl cDNA, 5μl 2x Master mix, 0.5μl Taqman primer, 3.5μl nuclease free water. All RT-qPCR experiments were performed in triplicate using the 7,500 Fast Real Time PCR machine (Applied Biosystems) and gene expression was quantified using the comparative C_T_ method, where each C_T_  value was first normalised to a respective ‘housekeeper’ and all samples were then standardised to a reference sample, final values were obtained using the formula 2^-∆∆CT [3]^. Taqman (ThermoFisher) primers used against the following mouse targets: HPRT1 (Mm03024075_m1), ACE (Mm00802048_m1), MME (Mm00485028_m1), COL4a3 (Mm00483669_m1) and COL4a4 (Mm00801574_m1), Hif1a1 (Mm00468869_m1) and Itgb3 (Mm00443980_m1).

# Supplementary Figures

**
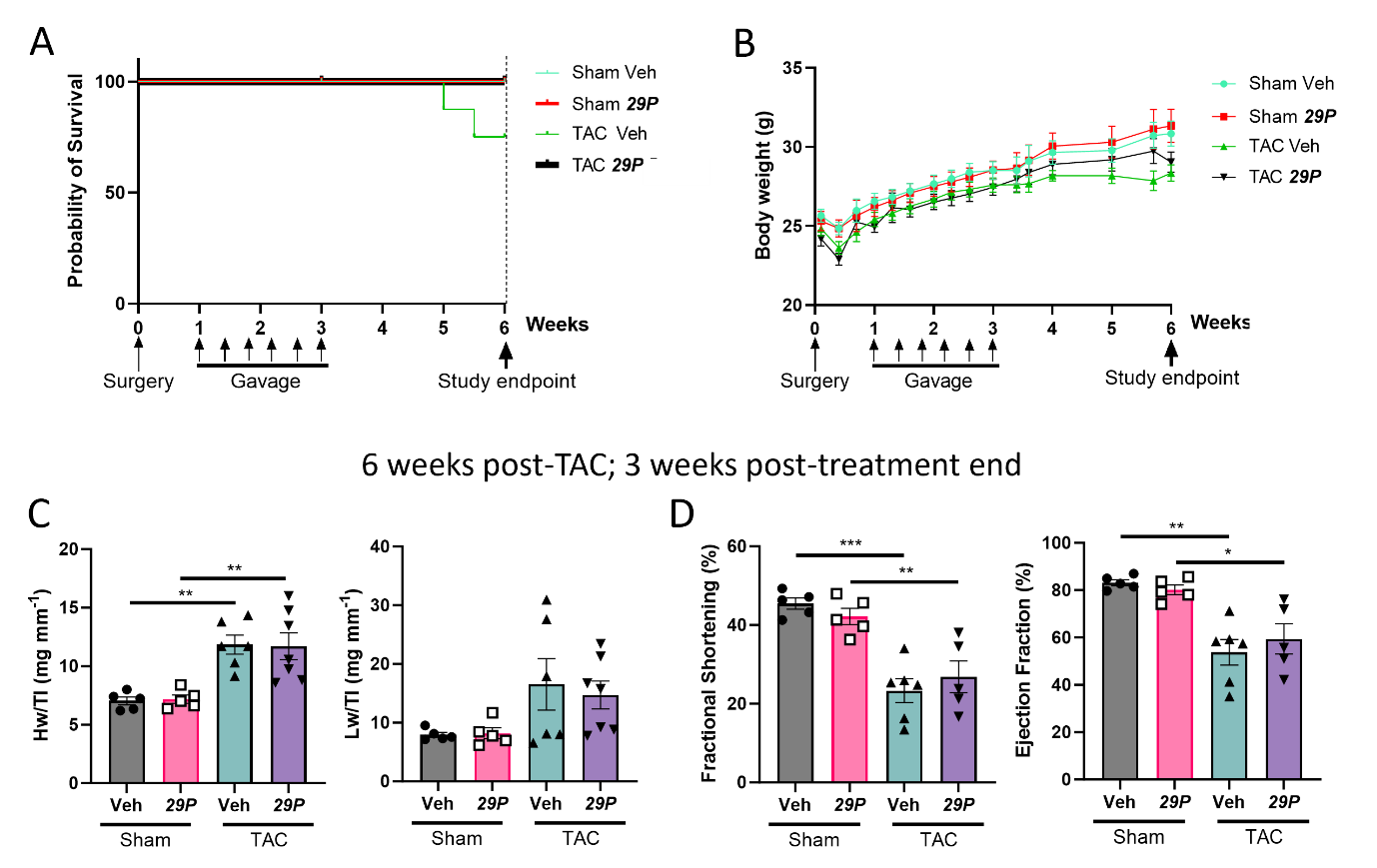
**

**Supplementary Figure 1: *29P* had no effect on survival or body weight but its cardioprotective effects are absent following treatment cessation**

**A** Survival study. No differences in survival between ***29P*** treated and Veh control mice: however, two Veh TAC mice were terminated before the end of the study due to reaching their humane endpoint (Sham Veh n=12, Sham ***29P*** n=12, TAC Veh n=15, TAC ***29P*** n=15). **B** Weight study. ***29P*** has no impact on weight. Data at 3 weeks: Sham Veh n=12, Sham ***29P*** n=12, TAC Veh n=13, TAC ***29P*** n=17; data at 6 weeks: Sham Veh n=5, Sham ***29P*** n=5, TAC Veh n=6, TAC ***29P*** n=7. **C** Morphometric parameters at 6 weeks post-TAC. Hw:Tl ratio is increased following TAC with no difference between treatment groups. Sham Veh n=5, Sham ***29P*** n=5, TAC Veh n=6, TAC ***29P*** n=7. **D** Representative M-mode echocardiographic images from 6 weeks post-TAC showed reduced cardiac function was present only in Veh TAC mice when compared to their respective Sham controls. Two ***29P***-treated TAC mice began to show signs of ill-health immediately before echocardiography at 6 weeks therefore echocardiography was not performed. Sham Veh n=5, Sham ***29P*** n=5, TAC Veh n=6, TAC ***29P*** n=5. **C, D** Two-way ANOVA with Tukey’s post-hoc multiple comparison test (*p<0.05, **p<0.01, ***p<0.001).

**
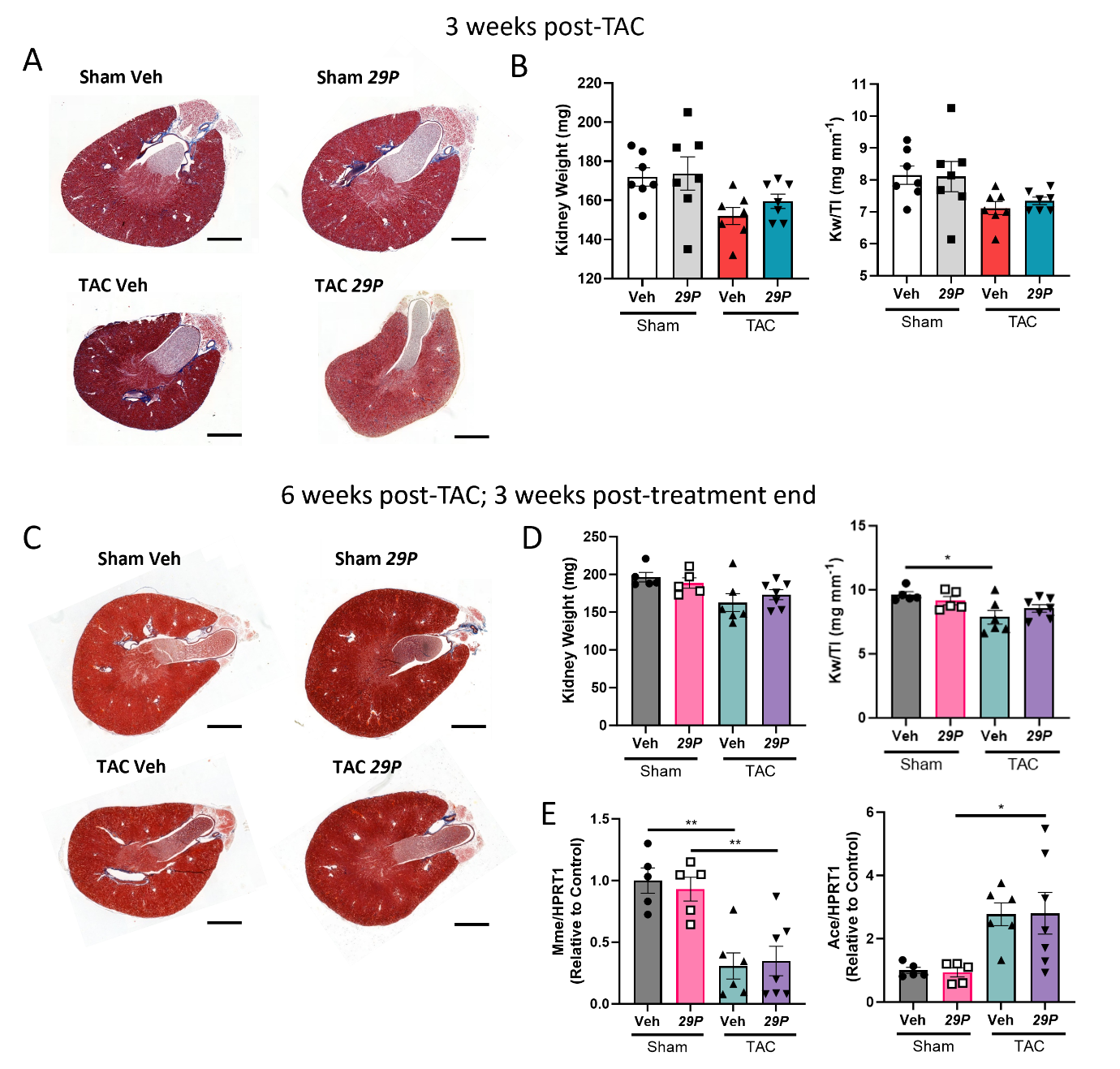
Supplementary Figure 2: TAC was associated with a partial reduction in kidney weight and kidney weight:tibia length ratio without changes to structural morphology**

Representative images of Masson’s Trichrome stained kidney sections at (**A**) 3 weeks and (**C**) 6-weeks post-TAC did not reveal any gross morphological changes. Scale bar = 1mm. Morphometric parameters at (**B**) 3-weeks and (**D**) 6-weeks post-TAC showed no significant differences by ***29P*** treatment. **E** RT-qPCR of *Ace* and *Mme* showed TAC induced transcript changes at 6-weeks post-TAC. **C** Sham Veh n=7, Sham ***29P*** n=7, TAC Veh n=7, TAC ***29P*** n=7. **D**, **E** Sham Veh n=5, Sham ***29P*** n=5, TAC Veh n=6, TAC ***29P*** n=7. All data analysed using two-way ANOVA with Tukey’s post-hoc multiple comparison test (*p<0.05, **p<0.01).

**
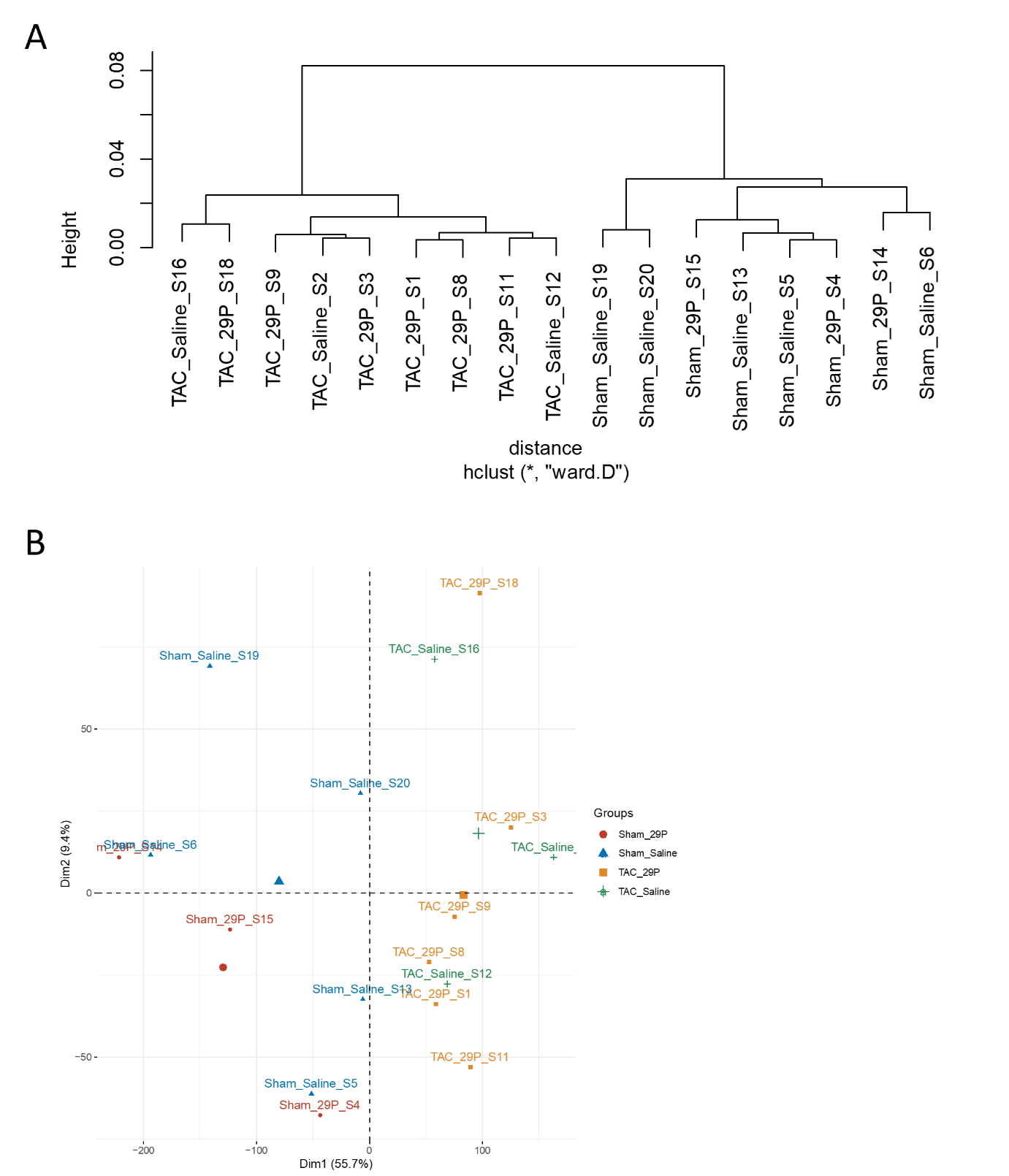
Supplementary Figure 3: Hierarchical clustering of RNA-Seq samples**

**A** Cluster dendrogram displaying hierarchical clustering of all samples subject to RNA-Seq showed separation of the TAC and Sham surgical groups. **B** PCA analysis of all samples showed distinct clusters based on surgery but not on treatment.

**
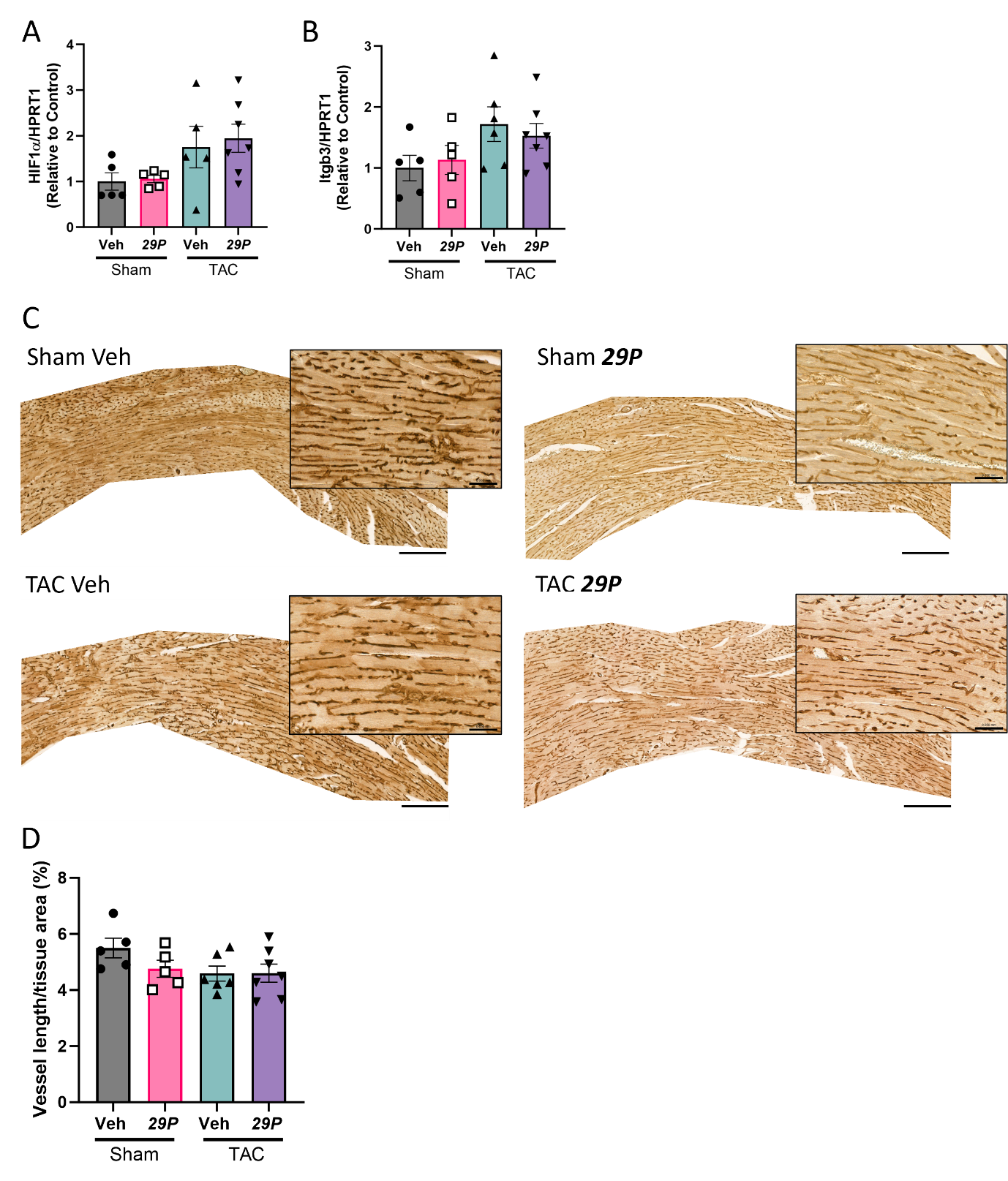
Supplementary Figure 4: At 6 weeks post-TAC there are no changes to the expression of key angiogenesis-related genes or microvessel complexity**

**A**, **B** RT-qPCR for *HIF-1α* (**A**) and *Itgb3* (**B**) showed no difference at 6 weeks post-TAC between groups. **C** Representative images from isolectin β4 stained tissue sections at 6-weeks post-TAC i.e., 3 weeks post treatment cessation. Scale bar = 200μm. Inset scale bar = 50μm. **D** The percentage of vessels per tissue area shows no change. Sham Veh n=5, Sham ***29P*** n=5, TAC Veh n=6, TAC ***29P*** n=7. Data analysed using Two-way ANOVA with Tukey’s post-hoc multiple comparison test.

**
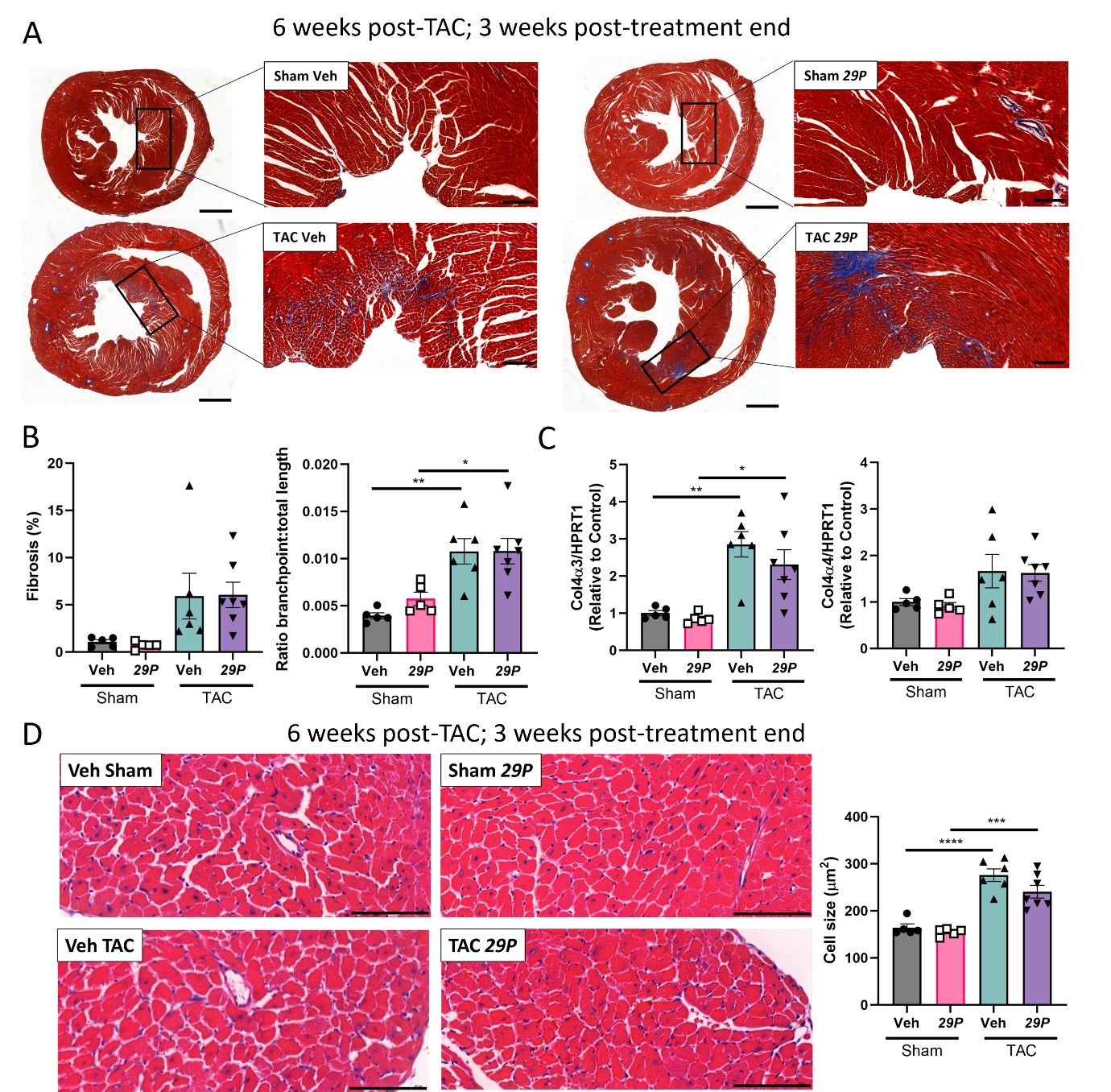
Supplementary Figure 5: At 6 weeks post-TAC, i.e. 3 weeks post-*29P* treatment cessation, *29P* is unable to maintain the partial cardioprotective effects conferred at 3 weeks post-TAC**

**A** Representative images of Masson’s trichrome stained tissue sections at 6 weeks post-TAC; 3 weeks post-treatment cessation. Scale bar(s): whole heart = 1mm, inset = 200μm. **B** TAC induced changes to percentage fibrosis and branchpoint but with no differences between treatment groups. **C** RT-qPCR of *Col4a3* and *Col4a4* showed no differences in expression level at the 6-week timepoint. **D** Representative images of H&E stained cardiac tissue sections and quantification of cardiomyocyte cell size showed TAC induced CSA increases but no treatment differences were present. Scale bar = 100μm. Sham Veh n=5, Sham ***29P*** n=5, TAC Veh n=6, TAC ***29P*** n=7. All data analysed using two-way ANOVA with Tukey’s post-hoc multiple comparison test (*p<0.05, **p<0.01, ****p<0.0001).

| Group comparisons | Upregulated DEGs | Downregulated DEGs |
| --- | --- | --- |
| Veh TAC vs. Veh Sham | 5722 | 477 |
| ***29P*** TAC vs. ***29P*** Sham | 6454 | 296 |
| ***29P*** Sham vs. Veh Sham | 221 | 721 |
| Veh TAC vs. ***29P*** TAC | 121 | 142 |

**Supplementary Table 1: Summary of the number of upregulated and downregulated genes identified following RNA-seq analysis**

Select group comparisons were performed to determine significant gene changes either following surgery alone, ***29P*** treatment alone or surgery with ***29P*** treatment.

# References

[1] E. Wershof, D. Park, D.J. Barry, R.P. Jenkins, A. Rullan, A. Wilkins, K. Schlegelmilch, I. Roxanis, K.I. Anderson, P.A. Bates, E. Sahai, A FIJI macro for quantifying pattern in extracellular matrix, Life Sci Alliance 4(3) (2021).

[2] I. Arganda-Carreras, R. Fernandez-Gonzalez, A. Munoz-Barrutia, C. Ortiz-De-Solorzano, 3D reconstruction of histological sections: Application to mammary gland tissue, Microsc Res Tech 73(11) (2010) 1019-29.

[3] K.J. Livak, T.D. Schmittgen, Analysis of relative gene expression data using real-time quantitative PCR and the 2(-Delta Delta C(T)) Method, Methods 25(4) (2001) 402-8.
